# Neoadjuvant therapy plus thulium laser transurethral bladder tumor resection for muscle-invasive bladder cancer

**DOI:** 10.14440/bladder.2024.0065

**Published:** 2025-06-05

**Authors:** Honglin Zhong, Wei He, Miao Mo, Shiyu Tong, Yinzhao Wang, Yuhang Wang, Xuhao Liu, Wenhao Zhu, Zhengchao Shen, Zhongfu Huang, Zhenyu Ou, Minfeng Chen

**Affiliations:** Department of Urology, National Clinical Research Center for Geriatric Disorders, Xiangya Hospital, Changsha, Hunan 410008, China

**Keywords:** Thulium, Muscle-invasive bladder cancer, Bladder preservation therapy, Transurethral resection of bladder tumor, Neoadjuvant therapy, Maintenance immunotherapy

## Abstract

**Background:**

Radical cystectomy (RC) is the standard treatment for muscle-invasive bladder cancer (MIBC). However, its high perioperative mortality and significant impact on quality of life have led many patients to opt for bladder-preserving approaches, which currently lack a standardized treatment protocol.

**Objective:**

This study evaluated the efficacy and feasibility of bladder-preserving therapy using thulium laser maximal transurethral resection of bladder tumors (TURBT) combined with neoadjuvant therapy and immunotherapy in MIBC patients.

**Methods:**

A retrospective analysis was conducted on 46 MIBC patients treated with neoadjuvant therapy followed by thulium laser maximal TURBT at a single center between January 2021 and October 2024. Patients had received neoadjuvant immunotherapy in combination with chemotherapy or antibody-drug conjugate before maximal thulium laser TURBT. Those who achieved a complete clinical response were allowed to pursue either bladder preservation or RC. Patients choosing bladder-preserving therapy were either given maintenance immunotherapy or put on observation. Bladder-intact disease-free survival (BIDFS) was assessed through Kaplan–Meier analysis, and Cox regression identified factors influencing BIDFS.

**Results:**

Among the 46 patients, 95.7% remained alive, and 82.6% demonstrated no evidence of cancer with bladder function preserved. The estimated 2-year BIDFS rate was 84.2%, and T stage and maintenance immunotherapy were identified as two independent predictors of BIDFS. Patients receiving immunotherapy were at a significantly reduced risk of recurrence compared to their counterparts under observation.

**Conclusion:**

Thulium laser maximal TURBT, combined with neoadjuvant therapy and maintenance immunotherapy, is a promising bladder-preserving approach that helps MIBC patients attain favorable BIDFS and quality of life.

## 1. Introduction

Bladder cancer represents the 10^th^ most common malignancy across the globe, with 573,278 new cases diagnosed in 2020, and a conspicuous male predominance.^1^ The condition is classified into non-muscle-invasive (NMIBC) and muscle-invasive bladder cancer (MIBC), with MIBC having a poorer prognosis. Over 90% of bladder cancer cases are urothelial carcinoma, which was the primary focus of this research. At present, the standard treatment includes cisplatin-based neoadjuvant chemotherapy, followed by radical cystectomy (RC) and pelvic lymph node dissection.^2^ However, RC carries significant perioperative risks, has long-term impacts on quality-of-life, and is unsuitable for some patients, highlighting the need for bladder-preserving alternatives.^2^-^4^

Transurethral resection of bladder tumors (TURBTs) is the primary surgical procedure for NMIBC.^5^ For MIBC patients opting for bladder preservation, maximal TURBT also plays a pivotal role in the treatment strategy. Comprehensive management includes neoadjuvant therapy before maximal TURBT and adjuvant therapy post-maximal TURBT. Various approaches are available for bladder preservation in MIBC. The most common option is trimodality therapy (TMT) for bladder preservation (maximal TURBT combined with chemotherapy and radiotherapy).^6^ While no large prospective randomized controlled trials compared the efficacy of RC and TMT, many high-quality retrospective analyses suggest that, in selected MIBC populations, TMT is not inferior to RC in terms of therapeutic efficacy.^7^-^9^ For example, a multicenter study published in 2023 showed that the 5-year disease-free survival for RC versus TMT was 73% (95% confidence interval [CI]: 69 – 77) versus 74% (95% CI: 69 – 79) with Inverse Probability of Treatment Weighting.^9^ In addition, with the development of neoadjuvant therapy for bladder cancer, many MIBC patients accomplished a complete response after the neoadjuvant therapy and subsequently chose bladder-preserving treatment.^10^ Cisplatin-based neoadjuvant chemotherapy has been shown to improve survival rates in MIBC.^11^,^12^ Approximately 30% of patients achieved pathological complete response following TURBT and neoadjuvant chemotherapy, highlighting its efficacy in eradicating micrometastatic disease.^11^,^13^ Immunotherapy and antibody-drug-conjugate drugs are expected to improve the effectiveness of neoadjuvant therapy in MIBC, enabling more patients to achieve a complete response and subsequently opt for bladder-preserving treatment rather than RC.^14^ Patients who achieve clinical complete response (cCR) after neoadjuvant therapy may also attain good disease-free survival by opting for bladder-preserving therapy instead of RC.^10^ However, there is no established standard adjuvant regimen for post-surgery bladder preservation for these patients, to whom available options included observation, immunotherapy, and chemoradiotherapy.^15^,^16^

With regard to bladder-preserving treatment for MIBC, TURBT is a vital component regardless of the integration of other non-surgical treatments. Nonetheless, conventional TURBT has limitations, including shallow resection depth, leading to incomplete tumor removal and necessitating re-TURBT. In addition, electrocautery can damage underlying tissues, compromising pathological staging accuracy and increasing risks such as obturator reflex and bladder perforation.^17^ Therefore, it is crucial to explore novel bladder-preserving TURBT surgical techniques to address these limitations. In clinical practice, thulium laser TURBT shows promise as an alternative to traditional TURBT.

In recent years, the thulium laser has become a widely used technology for *en bloc* resection of NMIBC. Compared to conventional TURBT, it has a higher absorption rate in water, stronger cutting ability, and reduced thermal damage to bladder tissues. Its continuous wave output allows for smooth incisions, effective tissue vaporization, and excellent hemostasis. Therefore, thulium laser surgery causes less intraoperative bleeding, has a lower incidence of obturator reflex and bladder perforation, and provides more accurate pathological staging of tumors.^17^-^19^ With precise control over the depth of resection, the thulium laser enables *en bloc* resection of diseased tissues, thereby enhancing surgeon’s ability to effectively excise suspicious lesions.

Given these advantages, our institution has employed maximal TURBT using a thulium laser for patients undergoing bladder preservation. Our research specifically focused on the thulium laser TURBT and exploited its potential to improve bladder preservation for MIBC. Our approach differed from the traditional TMT strategy in two key aspects. First, we employed the thulium laser TURBT as the surgical technique instead of conventional TURBT. Second, unlike the immediate implementation of bladder preservation therapy in the TMT regimen, our protocol administered neoadjuvant therapy first. Bladder preservation was then considered only for those patients who achieved cCR following neoadjuvant therapy, on the basis of a rigorous eligibility assessment. In addition, with our approach, post-operative management involved observation or immunotherapy, rather than concurrent chemoradiotherapy.

In this study, we improved surgical methods for patients undergoing bladder preservation treatment and the explored novel perioperative therapeutic strategies. To the best of our knowledge, this was the first investigation into the survival benefits of thulium laser therapy in bladder preservation for MIBC patients.

## 2. Materials and methods

### 2.1. Patient selection

We retrospectively reviewed 46 patients who had received neoadjuvant therapy plus thulium laser resection to preserve the bladder in our center from January 2021 to October 2024. The inclusion criteria were as follows: (i) age between 18 and 85 years, (ii) diagnosis of stage T2 to T3 urothelial MIBC as comfirmed by pre-operative computed tomography (CT)/magnetic resonance imaging (MRI) and post-operative pathology, (iii) availability of imaging data adequate for assessing the characteristics of tumors, (iv) detailed and complete data on perioperative treatment and outcomes, and (v) complete post-operative follow-up information. Patients who did not satisfy these criteria were excluded from the study.

**Figure 1:**
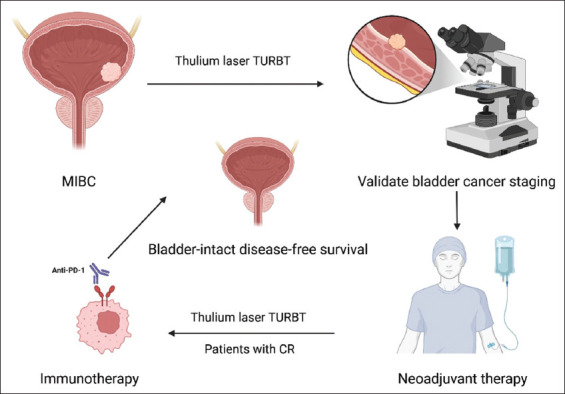
Treatment process Abbreviations: Anti-PD-1: Anti-programmed cell death 1; CR: Complete response; MIBC: Muscle-invasive bladder cancer; TURBT: Transurethral resection of bladder tumor.

For patients with suspected bladder cancer based on imaging, initial TURBT was performed using a thulium laser, and tissue samples were sent for pathological analysis. For confirmed MIBC cases, neoadjuvant therapy was given. The neoadjuvant regimen included immunotherapy (tislelizumab) combined with gemcitabine-plus-cisplatin chemotherapy lasting for three to four cycles and immunotherapy (toripalimab) in combination with disitamab vedotin treatment for four to six cycles. In patients with no significant residual tumor on post-neoadjuvant imaging, transurethral resection was again performed using a thulium laser to remove any visible tumor and/or tissue at the prior tumor site, with specimens submitted for pathological evaluation. The thulium laser was utilized to perform *en bloc* resection of the tumor bed tissue, including the mucosa and muscularis layers, aiming to reach the bladder serosal level. Patients achieving complete response either received maintenance immunotherapy with programmed cell death 1 (PD-1) inhibitors (tislelizumab or toripalimab) or were placed under observation (Figure 1). For patients who failed to achieve cCR, RC surgery was performed, and those who refused to receive RC were given chemoradiotherapy.

### 2.2. Surgical procedure

**Figure 2:**
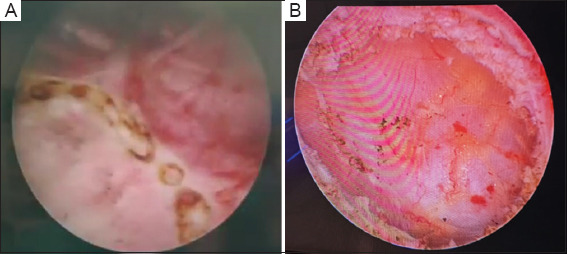
Intraoperative images. (A) Marking range before resection, and depth of tumor removal by thulium laser. The thulium laser can go deep into the tumor bed, reaching the bladder serosal layer. (B) The fat layer can be seen in the picture.

A thulium laser (SRMT1MAB, Raykeen, China) was used for the surgical procedure. The patients were placed in the lithotomy position under general anesthesia, with continuous irrigation using 0.9% sodium chloride. TURBT was performed according to standard protocol. Laser ablation was applied 0.5 – 1 cm from the tumor margin to delineate the resection boundary, ensuring complete tumor removal. Exposed blood vessels along this border were pre-coagulated. After undermining the mucosa, the submucosal layer of the tumor base was reached using the thulium laser, guided by a gentle push with a resectoscope beak. The fibrous connective tissue between the mucosal layer and the detrusor was identified. Resection depth was extended to the full thickness of the tumor bed once the serosa layer, indicated by visible fat particles and capillaries, was reached to prevent bladder rupture. The tumor and tumor bed were removed *en bloc* along anatomical planes. Surgical specimens were sent to the pathology department for diagnosis. Post-operative pathological staging after maximal TURBT confirmed negative margins and complete tumor excision. After surgery, a 22F Foley catheter was inserted, and bladder irrigation was discontinued once post-operative hematuria had resolved (Figure 2).

### 2.3. Post-operative and follow-up

Patients were checked every 3 months for the first 2 years in view of the high risk of recurrence and every 6 months thereafter with CT/MRI, cystoscopy, urine cytology, and routine blood and biochemical tests.

### 2.4. Endpoint

The primary endpoint was tumor recurrence, assessed using bladder-intact disease-free survival (BIDFS). BIDFS is the absence of bladder tumor recurrence, progression, or metastasis with preserved bladder function. Cox regression analysis was used to identify factors influencing BIDFS. In our study, cCR refers to pathologically diagnosed tumor T stage <1 (T0 and Ta) after neoadjuvant therapy, no malignant cells on urine cytology, and no definitive evidence of local or metastatic disease on cross-sectional imaging.

### 2.5. Statistical analysis

We used the Statistical Package for the Social Sciences version 27 for statistical analysis. Our primary outcome was BIDFS, and we performed a Kaplan–Meier analysis to analyze BIDFS in all patients. The 2-year BIDFS was obtained from the cumulative survival rate estimated in the survival analysis table. Clinical and pathological parameters were analyzed using Cox proportional hazards regression models, and univariate analysis was performed individually. For *p*<0.1, multivariate analysis was conducted using the stepwise forward selection method. For the interval estimation of the rate, we used bootstrapping to repeat the sampling 1,000 times and calculated the 95% CI. *p*<0.05 was considered statistically significant.

## 3. Results

After sufficient follow-up of these 46 patients, we obtained detailed data on patients who had undergone thulium laser treatment in our center from 2021 to 2024. The clinicopathological characteristics and post-operative conditions of the patients are shown in Table 1. A total of 46 MIBC patients were included in this study, with an average age of 67.85 years. Among them 39 were male (84.8%), accounting for the majority of the study population, and seven were female, constituting 15.2%, which is consistent with the notion that bladder cancer is more common in men.

**Table 1:** Baseline patient characteristics

Characteristics	Category	Data
Age (median [range], years)	Median	67.85 (49 – 84)
Sex	Male	39
	Female	7
Clinical stage	T2N0M0	39
	T2N1M0	3
	T3N0M0	4
Multiple tumors	Yes	26
	No	20
Immunotherapy	Yes	36
	No	10
Tumor recurrence	Yes	7
	No	39
Death	Yes	2
	No	44

Note: Data are presented as n, unless stated otherwise.

Among the 46 patients, 41 were staged as T2, and five as T3, with three having local lymph node metastases. All patients underwent at least two well-tolerated neoadjuvant therapy cycles, with no reports of adverse reactions above grade three, and without delays for the second TURBT due to neoadjuvant therapy. Following neoadjuvant therapy, the 46 patients achieved cCR, including 34 who reached T0 and 12 who reached Ta. After maximal TURBT with thulium laser, these patients declined radiotherapy. Ten patients chose observational management, while 36 opted for maintenance immunotherapy with PD-1 inhibitors (tislelizumab or toripalimab), involving between four and 18 treatment cycles.

**Figure 3:**
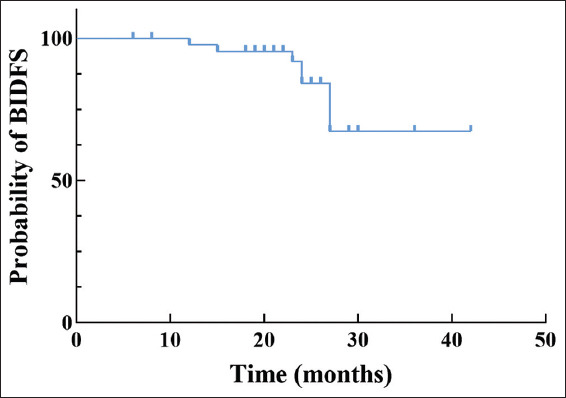
Kaplan–Meier curve of bladder-intact disease-free survival of patients

**Figure 4:**
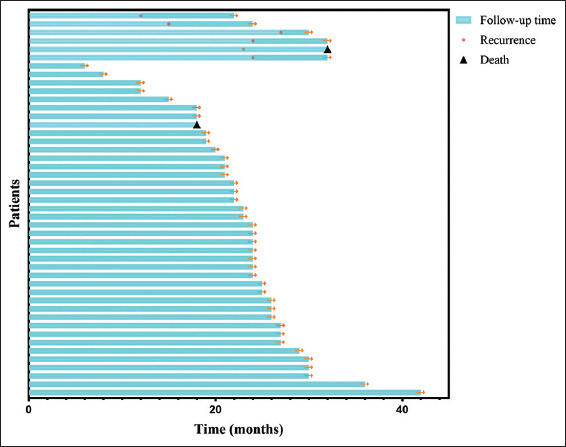
The swim plot of the clinical outcomes for each patient over time

The median follow-up duration in this study was 24 months (range: 6 – 42 months). As of October 2024, 44 patients were alive, with two recorded deaths. Among the cohort, seven patients suffered from recurrence, 38 remained disease-free, and one patient died of unrelated causes. The details of the seven recurrent patients are as follows: (i) one patient had MIBC and received RC, (ii) one patient developed brain metastasis and received intracranial tumor resection, (iii) one patient had lung metastasis and was given systemic treatment, and (iv) the rest had NMIBC in bladder and were subjected to transurethral laser resection plus subsequent bladder instillation of Bacillus Calmette–Guérin (Table 2). The overall 2-year BIDFS rate was 84.2%. The 2-year BIDFS rates for the observation and immunotherapy groups were 64.0% and 90.4%, respectively. The Kaplan–Meier curve for BIDFS is depicted in Figure 3, while Figure 4 presents a swim plot illustrating the clinical outcomes of each patient over time.

**Table 2:** Treatment patterns and recurrent patterns in recurrent patients

Recurrent patients	pTNM at diagnosis	Post-operative maintenance therapy	Recurrent cancer	Treatment after recurrence
1	T3N0M0	Observance	NMIBC (T1N0M0)	TURBT
2	T2N0M0	Observance	NMIBC (T1N0M0)	TURBT
3	T2N0M0	Observance	NMIBC (T1N0M0)	TURBT
4	T2N0M0	Immunotherapy	MIBC	RC
5	T3N0M0	Immunotherapy	BC brain metastasis	Intracranial tumor resection
6	T3N0M0	Immunotherapy	NMIBC (T1N0M0)	TURBT
7	T2N0M0	Immunotherapy	BC lung metastasis	Systemic treatment

Abbreviations: BC: Bladder cancer; MIBC: Muscle-invasive bladder cancer; NMIBC: Non-muscle-invasive bladder cancer; RC: Renal carcinoma; pTNM: Pathological tumor-node-metastasis staging; TURBT: Transurethral resection of bladder tumors.

Univariate and multivariate COX regression analyses showed that tumor T stage and the use of immunotherapy for maintenance treatment were independent influencing factors related to BIDFS (*p*<0.05), while other clinicopathological features, such as age, gender, and multiple tumors, exerted no significant effects on BIDFS. Multivariate COX regression analysis exhibited that the hazard ratio (HR) of T3 to T2 was 12.531 (95% CI: 2.038 – 77.050; *p*=0.006), indicating that the risk of bladder tumor recurrence or progression in patients with T3 was 12.531 times that of patients with T2. The HR of maintenance immunotherapy after thulium laser resection versus observation was 0.074 (95% CI: 0.009 – 0.624; *p*=0.017). This indicates that the risk of recurrence or progression in patients receiving maintenance immunotherapy is 0.074 times that of patients without receiving the treatment. The COX regression of univariate and multivariate analyses is shown in Table 3. The effects of tumor stage and post-operative maintenance immunotherapy on BIDFS were consistent with expectations. In contrast, other variables did not significantly impact BIDFS, which might be attributed to the limited sample size, insufficient statistical power, or the possibility that these variables had minimal influence on BIDFS.

**Table 3:** Cox proportional hazard regression analyses for bladder-intact disease-free survival

Parameter	Univariable analysis		Multivariable analysis	
	
	Hazard ratio (95% confidence interval)	*p*-value	Hazard ratio (95% confidence interval)	*p*-value
Age	0.948 (0.845 – 1.064)	0.363		
Gender				
Male	Reference			
Female	0.535 (0.064 – 4.494)	0.565		
Tumor stage				
T2	Reference			
T3	5.786 (1.289 – 25.980)	0.022	12.531 (2.038 – 77.050)	0.006
Node stage				
N0	Reference			
N1	0.044 (0 – 93005)	0.674		
Multiple tumors				
No	Reference			
Yes	0.685 (0.151 – 3.111)	0.624		
Immunotherapy				
No	Reference			
Yes	0.176 (0.029 – 1.057)	0.058	0.074 (0.009 – 0.624)	0.017

## 4. Discussion

This single-center study reported the preliminary findings of neoadjuvant therapy combined with transurethral thulium laser resection of bladder tumors as a bladder-preserving treatment for patients with MIBC.

Platinum-based neoadjuvant therapy has been widely used in clinical practice and recommended by guidelines.^2^ With the development of drug treatment for bladder cancer, including the widespread application of immunotherapy and antibody-drug conjugate drugs, more MIBC patients are receiving neoadjuvant therapy, and the rate of patients achieving a complete response after treatment has been on the rise.^10^,^20^ While most guidelines recommend RC surgery after neoadjuvant therapy for MIBC patients, many patients who achieve a complete response choose bladder-preserving treatment due to the higher risk of RC surgery and the greater impact on the patient’s quality of life after surgery.^2^,^10^ At present, no unified bladder-preserving treatment plan is available for such patients, including observation, immunotherapy, and chemoradiotherapy. There is still a lack of high-level clinical data that help determine which plan is more effective. In the TMT bladder-preserving treatment model, radiotherapy plays a key role, and this model has also been reported in many clinical reports.^15^,^21^,^22^ However, for patients who have achieved a complete response with neoadjuvant therapy, the necessity for a radiotherapy in the subsequent bladder-preserving treatment is still inconclusive.

Included in our study were MIBC patients who achieved cCR after neoadjuvant therapy and refused cystectomy and radiotherapy. Most of these patients chose maintenance immunotherapy in the subsequent bladder preservation process, and a few opted for close observation. At a median follow-up of 24 months, 95.7% of patients were alive, and 82.6% were alive with good bladder function and without evidence of cancer recurrence. The 2-year BIDFS rate was 84.2%. The 2-year BIFDS in the observation and immunotherapy groups were 64.0% and 90.4%, respectively. The difference in the 2-year BIDFS between immunotherapy and observation therapy could be ascribed to the presence of minimal residual disease. Although imaging and pathological examinations indicated that there was no visible tumor residue after TURBT, there might still be microscopic residual lesions. Immunotherapy can activate the body’s immune system to identify and eliminate these residual cancer cells, thereby precluding recurrence or metastasis.^23^ In addition, immunotherapy (such as PD-1/programmed death-ligand 1 inhibitors) activates immune effector cells, such as T cells, enabling the body to generate immune memory against tumor antigens. Even if the primary tumor is completely removed, the “memory effect” of the immune system remains, and it can respond quickly to monitor and eliminate tumor cells when they reappear.^24^ These data are similar to those observed in other bladder-preserving combined therapies.^25^-^27^ In 2014, a study on maximal TURBT in combination with internal iliac artery chemotherapy and intravesical instillation for the treatment of MIBC patients showed that the 2-year disease-free survival rate of 62 patients could arrive at 77.8%.^25^ A 2024 study comparing bladder-preserving therapy with RC therapy involving 1,432 patients revealed that the 2-year disease-free survival rate of patients treated with bladder-preserving therapy was 61.5%.^26^ A retrospective multicenter study of concurrent chemoradiotherapy for non-metastatic MIBC in 2022 demonstrated that the 2-year bladder-intact event-free survival rate of 240 MIBC patients was 75%.^28^ The latest TMT therapy evidence showed that the 5-year disease-free survival rate of the TMT strategy for bladder preservation treatment was 74%.^9^ These findings are comparable to the 2-year BIDFS rate of 84.2% observed in our study. Notably, our 2-year BIDFS appears more favorable, potentially reflecting the survival benefits associated with thulium laser treatment. In addition, the patients included were mainly in the T2 stage, and these patients had a better bladder preservation effect. As the follow-up of our study is extended and the sample size increases, the BIDFS may further drop, and the 5-year BIDFS of the immunotherapy group in our study may be close to the results of TMT.

Maximal TURBT is a critical step in the bladder preservation strategy for MIBC. Complete removal of tumor tissues and suspicious lesions is essential to maximizing therapeutic efficacy and reducing the risk of recurrence.^2^,^15^,^29^ TURBT can be performed using traditional electrosurgical resection or laser resection techniques. The thulium laser is a popular laser used in recent years. Compared with traditional electrosurgical resection, thulium lasers have many advantages in urological surgery, especially in tumor resection. First, thulium lasers have higher cutting accuracy and better tissue selectivity, which minimizes damage to surrounding healthy tissues during surgery, thereby protecting organ function. Second, thanks to its lower penetration depth, thulium lasers can achieve more thorough full-thickness resection on the tumor bed, theoretically significantly reducing the probability of tumor recurrence. In addition, thulium lasers can attain better hemostatic effects, which can lower the risk of intraoperative bleeding and improve surgical safety. Our study showed that the use of thulium laser technology can remove the tumor bed to a greater extent, thus potentially improving patient prognosis.^17^,^18^

In recent years, immune checkpoint inhibitors have been developed and approved for clinical use and have demonstrated strong anti-tumor effects on various tumors, including urothelial carcinoma.^30^ Although the three-stage bladder-preserving treatment is a classic bladder-preserving alternative,^2^,^7^ we used neoadjuvant therapy in combination with thulium laser transurethral bladder tumor resection, followed by post-operative immunotherapy as a maintenance bladder-preserving treatment for MIBC. Multiple clinical studies have shown that PD-1 immune agents improve the prognosis of bladder cancer patients at various disease states. For example, it is used for bladder-preserving treatment of high-risk patients unresponsive to Bacillus Calmette–Guérin,^31^ an adjuvant treatment for high-risk muscle-invasive urothelial carcinoma after radical surgery,^32^ and for maintenance treatment of advanced or metastatic urothelial carcinoma.^33^ In our center, we utilized the PD-1 inhibitors, tislelizumab or toripalimab, which are widely used in China. Clinical trials have shown that tislelizumab has a significant clinical benefit with a manageable safety profile in Asian patients with locally advanced or metastatic urothelial carcinoma. Tislelizumab combined with gemcitabine plus cisplatin chemotherapy as neoadjuvant therapy can improve the efficacy of neoadjuvant treatment of MIBC. Compared to neoadjuvant immunotherapy or neoadjuvant chemotherapy alone, this combination therapy can achieve the highest complete response rate and pathological downstaging rate.^29^ Toripalimab is also used in the treatment of urothelial carcinoma. A multicenter phase II clinical trial investigated the efficacy of toripalimab in patients with metastatic urothelial carcinoma who had failed standard therapy, and concluded that toripalimab has good clinical activity and controllable safety in the treatment of metastatic urothelial carcinoma.^34^ On the basis of our retrospective clinical observation, we are led to conclude that the use of tislelizumab/toripalimab exerts a good maintenance treatment effect on patients undergoing bladder preservation treatment. There has been controversy over the use of platinum-based chemotherapy after surgery for high-risk MIBC patients. A systematic review and meta-analysis have shown that adjuvant cisplatin chemotherapy can benefit MIBC patients in terms of overall survival.^2^ Many studies use chemotherapy drugs as radiotherapy sensitizers for bladder preservation. However, the chemotherapeutics have numerous adverse effects.^6^ Immunotherapy is emerging as a promising approach in the bladder preservation treatment of MIBC. Although its role is still under investigation, its growing application highlights its increasing significance in this field. Ongoing clinical trials are integrating immunotherapy with chemoradiotherapy to evaluate its impact on patient survival.^6^ Early findings from these studies indicated immunotherapy which had the therapeutic potential and suggested that it may enhance treatment outcomes, further supporting bladder-preserving strategies for MIBC patients.^10^ Compared to patients receiving platinum-based chemotherapy, those who use immunotherapy for maintenance have lower renal function requirements, a lower incidence of serious adverse reactions, and better tolerance. Hence, more patients prefer immunotherapy.

For MIBC bladder preservation treatment, it is crucial to select the right population. The preferred patients for traditional TMT model are those with small tumors (<5 cm), with a unifocal lesion, without microscopic remnants after TURBT, with no ureteral obstruction or hydronephrosis, without association with carcinoma *in situ* (accurately diagnosed by biopsy of suspicious areas and histological analysis of previous TURBT), and with no evidence of pelvic lymph node disease.^35^,^36^ About 15% of MIBC patients are estimated to be suitable for bladder preservation treatment.^37^ Bladder preservation treatment has good application prospects and is worthy of further research. MIBC patients receiving neoadjuvant therapy may not be suitable for bladder preservation therapy before the treatment, but after neoadjuvant therapy, if the patient responds completely, bladder preservation can be considered. After neoadjuvant therapy, more patients who achieve cCR choose bladder preservation treatment. Our study aimed to explore the efficacy of bladder preservation in this population. Our preliminary findings showed that it is feasible for MIBC patients who achieve cCR after neoadjuvant therapy to receive laser resection and immunotherapy for bladder preservation.

While study used thulium laser TURBT and immunotherapy to preserve the bladder in patients with MIBC who achieve cCR and yielded promising results, it is subject to several limitations. First, this was a single-center retrospective study without a control group. Without a randomized controlled trial, it is difficult to compare the differences between other bladder-preserving treatments (such as traditional wire-loop electrode maximal bladder tumor resection) and bladder-preserving treatment after thulium laser maximal tumor resection. It is also hard to compare the differences between RC and bladder-preserving treatment after thulium laser maximal tumor resection. Second, our results could not be extrapolated to all MIBC patients, but only to a small number of patients who meet strict selection criteria. The extent of TURBT is difficult to define and may depend on the physician. Therefore, this treatment may only be feasible in centers experienced with urological tumors. In addition, the number of patients included in this study was small, and a certain degree of heterogeneity among the patients was inevitable. This resulted in the inability to accurately estimate the HR of covariates when conducting statistical analysis, and the statistical power was insufficient, which would lead to certain biases. Under the same treatment conditions, individual differences in patients’ responsiveness to drugs and different molecular features of tumors may affect disease-free survival. Furthermore, our follow-up time was not long enough, and a longer follow-up is needed to observe the bladder-preserving effect on long-term basis. Therefore, multicenter retrospective cohort studies, prospective cohort studies, or clinical trials are warranted in future for further validation of our findings.

Genetic biomarkers are being studied to determine which patients can benefit from bladder preservation. DNA damage and repair genes have been gaining attention in recent years. A series of changes in genes related to DNA damage and repair, such as *ATM*, *RB1*, *FANCC*, and *ERCC2*, have been shown to be associated with the prognosis of MIBC after chemotherapy.^6^,^38^,^39^ Although research is still lacking on these related genes and prognosis in immunotherapeutic regimens, this suggests that, in the future, for MIBC patients who wish to undergo bladder-preserving treatment, the genetic changes in the tumor can be detected to select appropriate treatment options, thereby achieving personalized treatment and precision medicine. In addition, more methods, such as immune microenvironment typing and imaging genomics, must be developed to accurately screen the MIBC population suitable for bladder preservation.

## 5. Conclusion

To the best of our knowledge, our study was the first to investigate the effect of neoadjuvant therapy combined with thulium laser resection and post-operative maintenance immunotherapy on patients with MIBC. Our research showed that this approach is safe and feasible. This single-center study provided preliminary evidence that can inform subsequent research, and future controlled studies are needed for further validation.

## Data Availability

Data will be made available on reasonable request from the corresponding author.

## References

[1] Sung H Ferlay J Siegel RL Global cancer statistics 2020:Globocan estimates of incidence and mortality worldwide for 36 cancers in 185 countries. CA Cancer J Clin 2021;71 (3):209–249. doi:10.3322/caac.21660 33538338 10.3322/caac.21660

[2] Alfred Witjes J Max Bruins H Carrión A European association of urology guidelines on muscle-invasive and metastatic bladder cancer:Summary of the 2023 guidelines. Eur Urol 2024;85 (1):17–31. doi:10.1016/j.eururo.2023.08.016 37858453 10.1016/j.eururo.2023.08.016

[3] Quek ML Stein JP Daneshmand S A critical analysis of perioperative mortality from radical cystectomy. J Urol 2006;175 (3 Pt 1):886–9. discussion 889-90 doi:10.1016/s0022-5347(05)00421-0 16469572 10.1016/S0022-5347(05)00421-0

[4] Stein JP Lieskovsky G Cote R Radical cystectomy in the treatment of invasive bladder cancer:Long-term results in 1,054 patients. J Clin Oncol 2001;19 (3):666–675. doi:10.1200/jco.2001.19.3.666 11157016 10.1200/JCO.2001.19.3.666

[5] Babjuk M Burger M Compérat EM European association of urology guidelines on non-muscle-invasive bladder cancer (TaT1 and carcinoma in situ) - 2019 update. Eur Urol 2019;76 (5):639–657. doi:10.1016/j.eururo.2019.08.016 31443960 10.1016/j.eururo.2019.08.016

[6] Niglio SA Purswani JM Schiff PB Organ preservation in muscle-invasive urothelial bladder cancer. Curr Opin Oncol 2024;36 (3):155–163. doi:10.1097/cco.0000000000001038 38573204 10.1097/CCO.0000000000001038

[7] Efstathiou JA Spiegel DY Shipley WU Long-term outcomes of selective bladder preservation by combined-modality therapy for invasive bladder cancer:The MGH experience. Eur Urol 2012;61 (4):705–711. doi:10.1016/j.eururo.2011.11.010 22101114 10.1016/j.eururo.2011.11.010

[8] Büchser D Zapatero A Rogado J Long-term outcomes and patterns of failure following trimodality treatment with bladder preservation for invasive bladder cancer. Urology 2019;124 183–190. doi:10.1016/j.urology.2018.07.058 30266376 10.1016/j.urology.2018.07.058

[9] Zlotta AR Ballas LK Niemierko A Radical cystectomy versus trimodality therapy for muscle-invasive bladder cancer:A multi-institutional propensity score matched and weighted analysis. Lancet Oncol 2023;24 (6):669–681. doi:10.1016/s1470-2045(23)00170-5 37187202 10.1016/S1470-2045(23)00170-5

[10] Galsky MD Daneshmand S Izadmehr S Gemcitabine and cisplatin plus nivolumab as organ-sparing treatment for muscle-invasive bladder cancer:A phase 2 trial. Nat Med 2023;29 (11):2825–2834. doi:10.1038/s41591-023-02568-1 37783966 10.1038/s41591-023-02568-1 PMC10667093

[11] Grossman HB Natale RB Tangen CM Neoadjuvant chemotherapy plus cystectomy compared with cystectomy alone for locally advanced bladder cancer. N Engl J Med 2003;349 (9):859–866. doi:10.1056/NEJMoa022148 12944571 10.1056/NEJMoa022148

[12] Griffiths G Hall R Sylvester R Raghavan D Parmar MK International phase III trial assessing neoadjuvant cisplatin, methotrexate, and vinblastine chemotherapy for muscle-invasive bladder cancer:Long-term results of the BA06 30894 trial. J Clin Oncol 2011;29 (16):2171–2177. doi:10.1200/jco.2010.32.3139 21502557 10.1200/JCO.2010.32.3139 PMC3107740

[13] Flaig TW Tangen CM Daneshmand S A randomized phase II study of coexpression extrapolation (COXEN) with neoadjuvant chemotherapy for bladder cancer (SWOG S1314;NCT02177695). Clin Cancer Res 2021;27 (9):2435–2441. doi:10.1158/1078-0432.Ccr-20-2409 33568346 10.1158/1078-0432.CCR-20-2409 PMC8219246

[14] Wen F Lin T Zhang P Shen Y RC48-ADC combined with tislelizumab as neoadjuvant treatment in patients with HER2-positive locally advanced muscle-invasive urothelial bladder cancer:A multi-center phase Ib/II study (HOPE-03). Front Oncol 2023;13 1233196 doi:10.3389/fonc.2023.1233196 38269021 10.3389/fonc.2023.1233196 PMC10806139

[15] Geavlete P Georgescu D Florea I Second transurethral resection and adjuvant radiotherapy in conservative treatment of pT2N0M0 bladder tumors. Eur Urol 2003;43 (5):499–504. doi:10.1016/s0302-2838(03)00098-8 12705994 10.1016/s0302-2838(03)00098-8

[16] Merseburger AS Kuczyk MA The value of bladder-conserving strategies in muscle-invasive bladder carcinoma compared with radical surgery. Curr Opin Urol 2007;17 (5):358–362. doi:10.1097/MOU.0b013e3282c4afa0 17762631 10.1097/MOU.0b013e3282c4afa0

[17] Liu Z Zhang Y Sun G Comparison of thulium laser resection of bladder tumors and conventional transurethral resection of bladder tumors for non-muscle-invasive bladder cancer. Urol Int 2022;106 (2):116–121. doi:10.1159/000514042 33784709 10.1159/000514042

[18] Assem A Kassem A Sherif M Lotfi A Abdelwahed M Safety, feasibility, and quality of thulium laser en-bloc resection for treatment of non-muscle invasive bladder cancer. Int Urol Nephrol 2023;55 (12):3103–3109. doi:10.1007/s11255-023-03752-5 37639155 10.1007/s11255-023-03752-5 PMC10611837

[19] Migliari R Buffardi A Ghabin H Thulium laser endoscopic en bloc enucleation of nonmuscle-invasive bladder cancer. J Endourol 2015;29 (11):1258–1262. doi:10.1089/end.2015.0336 26102556 10.1089/end.2015.0336

[20] Patel VG Oh WK Galsky MD Treatment of muscle-invasive and advanced bladder cancer in 2020. CA Cancer J Clin 2020;70 (5):404–423. doi:10.3322/caac.21631 32767764 10.3322/caac.21631

[21] Mak RH Hunt D Shipley WU Long-term outcomes in patients with muscle-invasive bladder cancer after selective bladder-preserving combined-modality therapy:A pooled analysis of Radiation Therapy Oncology Group protocols 8802, 8903, 9506, 9706, 9906, and 0233. J Clin Oncol 2014;32 (34):3801–3809. doi:10.1200/jco.2014.57.5548 25366678 10.1200/JCO.2014.57.5548 PMC4239302

[22] Ploussard G Daneshmand S Efstathiou JA Critical analysis of bladder sparing with trimodal therapy in muscle-invasive bladder cancer:A systematic review. Eur Urol 2014;66 (1):120–137. doi:10.1016/j.eururo.2014.02.038 24613684 10.1016/j.eururo.2014.02.038

[23] Zhang R Zang J Jin D Urinary tumor DNA MRD analysis to identify responders to neoadjuvant immunotherapy in muscle-invasive bladder cancer. Clin Cancer Res 2023;29 (20):4040–4046. doi:10.1158/1078-0432.Ccr-23-0513 37535065 10.1158/1078-0432.CCR-23-0513

[24] Lahiri A Maji A Potdar PD Lung cancer immunotherapy:Progress, pitfalls, and promises. Mol Cancer 2023;22 (1):40 doi:10.1186/s12943-023-01740-y 36810079 10.1186/s12943-023-01740-y PMC9942077

[25] Li J Wang Q Xiao B Zhang X Effect of internal iliac artery chemotherapy after transurethral resection of bladder tumor for muscle invasive bladder cancer. Chin J Cancer Res 2014;26 (5):558–563. doi:10.3978/j.issn.1000-9604.2014.10.05 25400421 10.3978/j.issn.1000-9604.2014.10.05 PMC4220261

[26] Brück K Meijer RP Boormans JL Disease-free survival of patients with muscle-invasive bladder cancer treated with radical cystectomy versus bladder-preserving therapy:A nationwide study. Int J Radiat Oncol Biol Phys 2024;118 (1):41–49. doi:10.1016/j.ijrobp.2023.07.027 37517601 10.1016/j.ijrobp.2023.07.027

[27] Cho SW Lim SH Kwon GY Neoadjuvant cisplatin-based chemotherapy followed by selective bladder preservation chemoradiotherapy in muscle-invasive urothelial carcinoma of the bladder:Post hoc analysis of two prospective studies. Cancer Res Treat 2024;56 (3):893–897. doi:10.4143/crt.2024.015 38374699 10.4143/crt.2024.015 PMC11261190

[28] De Ruiter BM Van de Kamp MW Van Steenbergen JPZ A multicenter retrospective cohort series of muscle-invasive bladder cancer patients treated with definitive concurrent chemoradiotherapy in daily practice. Eur Urol Open Sci 2022;39 7–13. doi:10.1016/j.euros.2022.02.010 35528785 10.1016/j.euros.2022.02.010 PMC9068732

[29] Hu J Chen J Ou Z Neoadjuvant immunotherapy, chemotherapy, and combination therapy in muscle-invasive bladder cancer:A multi-center real-world retrospective study. Cell Rep Med 2022;3 (11):100785 doi:10.1016/j.xcrm.2022.100785 36265483 10.1016/j.xcrm.2022.100785 PMC9729796

[30] Powles T Durán I Van der Heijden MS Atezolizumab versus chemotherapy in patients with platinum-treated locally advanced or metastatic urothelial carcinoma (IMvigor211):A multicentre, open-label, phase 3 randomised controlled trial. Lancet 2018;391 (10122):748–757. doi:10.1016/s0140-6736(17)33297-x 29268948 10.1016/S0140-6736(17)33297-X

[31] Balar AV Kamat AM Kulkarni GS Pembrolizumab monotherapy for the treatment of high-risk non-muscle-invasive bladder cancer unresponsive to BCG (KEYNOTE-057):An open-label, single-arm, multicentre, phase 2 study. Lancet Oncol 2021;22 (7):919–930. doi:10.1016/s1470-2045(21)00147-9 34051177 10.1016/S1470-2045(21)00147-9

[32] Bajorin DF Witjes JA Gschwend JE Adjuvant nivolumab versus placebo in muscle-invasive urothelial carcinoma. N Engl J Med 2021;384 (22):2102–2114. doi:10.1056/NEJMoa2034442 34077643 10.1056/NEJMoa2034442 PMC8215888

[33] Powles T Park SH Voog E Avelumab maintenance therapy for advanced or metastatic urothelial carcinoma. N Engl J Med 2020;383 (13):1218–1230. doi:10.1056/NEJMoa2002788 32945632 10.1056/NEJMoa2002788

[34] Sheng X Chen H Hu B Safety, efficacy, and biomarker analysis of toripalimab in patients with previously treated advanced urothelial carcinoma:Results from a multicenter phase II Trial POLARIS-03. Clin Cancer Res 2022;28 (3):489–497. doi:10.1158/1078-0432.Ccr-21-2210 34740921 10.1158/1078-0432.CCR-21-2210

[35] Smith AB Deal AM Woods ME Muscle-invasive bladder cancer:Evaluating treatment and survival in the National cancer data base. BJU Int 2014;114 (5):719–726. doi:10.1111/bju.12601 24325202 10.1111/bju.12601

[36] James ND Hussain SA Hall E Radiotherapy with or without chemotherapy in muscle-invasive bladder cancer. N Engl J Med 2012;366 (16):1477–1488. doi:10.1056/NEJMoa1106106 22512481 10.1056/NEJMoa1106106

[37] Smith ZL Christodouleas JP Keefe SM Malkowicz SB Guzzo TJ Bladder preservation in the treatment of muscle-invasive bladder cancer (MIBC):A review of the literature and a practical approach to therapy. BJU Int 2013;112 (1):13–25. doi:10.1111/j.1464-410X.2012.11762.x 23356411 10.1111/j.1464-410X.2012.11762.x

[38] Taber A Christensen E Lamy P Molecular correlates of cisplatin-based chemotherapy response in muscle invasive bladder cancer by integrated multi-omics analysis. Nat Commun 2020;11 (1):4858 doi:10.1038/s41467-020-1⇀-0 32978382 10.1038/s41467-020-18640-0 PMC7519650

[39] Miron B Hoffman-Censits JH Anari F Defects in DNA repair genes confer improved long-term survival after cisplatin-based neoadjuvant chemotherapy for muscle-invasive bladder cancer. Eur Urol Oncol 2020;3 (4):544–547. doi:10.1016/j.euo.2020.02.003 32165095 10.1016/j.euo.2020.02.003 PMC7689684

